# Redefining Cancer Care in India: Dr. Viswanathan Shanta's Lasting Contributions and Its Enduring Impact

**DOI:** 10.7759/cureus.68334

**Published:** 2024-08-31

**Authors:** Navaudhayam Ranganathan, Anu Agrawal, Winsome Kumar, Anirudh Singh, Arun Yadav

**Affiliations:** 1 Radiation Oncology, All India Institute of Medical Sciences, New Delhi, New Delhi, IND; 2 Radiation Oncology, Vardhman Mahavir Medical College and Safdarjung Hospital, New Delhi, IND

**Keywords:** medical innovation, historical vignette, medical stories, biographies, historical vignettes

## Abstract

Dr. V. Shanta (1927-2021) made priceless contributions that significantly changed advanced cancer care in India. Trained at Madras Medical College and further trained in oncology internationally, she transformed the Cancer Institute in Chennai from a 12-bed facility into a leading cancer care institute of global repute. Dr. Shanta championed early cancer detection and sought to dismantle the stigma surrounding the disease, particularly in rural communities. Under her leadership, the institute introduced pioneering technologies and established India's first pediatric oncology institute. She was instrumental in creating comprehensive cancer registries that informed national health policies. Her dedication to holistic, patient-oriented care and her commitment to equitable healthcare earned her numerous accolades, including India's highest civilian awards such as the Padma Vibhushan and Padma Bhushan and Asia's premier prize the Ramon Magsaysay Award. Her work remains a cornerstone of oncology in India, continuing to inspire future generations of medical professionals.

## Introduction and background

On the eve of the 78th Independence Day, as a young radiation oncologist and as a society as a whole, it is important to reflect on the contributions of those who have shaped the landscape of cancer care in our nation. One such luminary is a visionary who dedicated her entire life to transforming the perception of cancer, tirelessly treating patients from all sections of society with unparalleled care and empathy. She not only standardized cancer treatment protocols but also established a world-renowned cancer institute that stands as a beacon of excellence. Her journey of hard work, passion, and dedication continues to inspire and motivate us to strive for excellence in our own paths.

Formative years and educational path

In the British-ruled Madras Presidency, on 11th March 1927, Dr. Viswanathan Shanta was born during the pre-independent era. During the pre-independent era, in 1901, the female literacy rate in India was as low as 0.6%, as the condition of females was marred by sati, child marriage, female infanticide, polygamy, and patriarchy [1]. She had to fight an uphill battle from the beginning with the general perception of society in terms of females. Although she was born into a family of two well-renowned Nobel laureates, C. V. Raman and S. Chandrasekhar [2], aspirations of becoming a doctor at a very young age of 12 years were kindled by the likes of Dr. Muthulakshmi Reddy (first female medical graduate in the country) and Lady Dufferin [3]. She secured her bachelor's degree in medicine in 1949, diploma in gynecology and obstetrics in 1952, and doctor of medicine in obstetrics and gynecology in 1955. Later on, she was trained in advanced cancer care in Toronto in 1957, followed by bone marrow transplantation training in 1958 from the United Kingdom.

Career

Presently known as Adyar Cancer Institute, the Cancer Institute was established by Dr. Muthulakshmi Reddy (the world's first female vice president of a legislature) in 1954; at the same time, she had just finished her post graduation. After clearing the public service examination, she was posted at the Women and Children Hospital. Then, she joined as an honorary staff at the Cancer Institute under Dr. Krishnamurthi, which was a little, one-story cottage hospital with 12 beds and two physicians. She moved onto the campus on April 13, 1955, as an honorary staff member. She dedicated her entire life to the betterment of cancer patients, especially patients belonging to lower economic strata. She continuously fought against the perception of cancer disease among the society until she passed away on January 19, 2021.

## Review

Creating impact: Expanding cancer care accessibility in a resource-limited setting

In a country where out-of-pocket expenditure for health remains alarmingly high at almost 47.1% [4] and 37% [5] in terms of US dollars according to World Bank data and where healthcare affordability continues to be a challenge, it becomes imperative to highlight the exceptional contributions of institutions that bridge this gap. The Cancer Institute stands as a testament to compassionate healthcare, having been established as a charitable, not-for-profit hospital. Despite facing significant financial challenges, the institute has remained steadfast in its commitment to providing care for all. Remarkably, 60% of its patients receive free treatment, with the hospital also covering travel allowances and providing free accommodation for those in need.

This remarkable institution, under Dr. V. Shanta's visionary leadership, grew from a modest 12-bed hospital into a cutting-edge cancer care institute with 650 beds. Over six decades, she transformed it into a research institute of both national and international renown, serving over 15,000 underprivileged patients annually. Her unwavering dedication ensured that those who could not afford treatment were offered reduced-cost options, making advanced cancer care accessible to the most vulnerable sections of society. The institute's legacy is not just in its world-class facilities but also in the hope and dignity it has restored to countless lives.

A legacy of firsts

The legacy of pioneering advancements in cancer care and research is deeply etched in the history of the Cancer Institute. With unwavering support from the Atomic Energy Regulatory Board, the institute achieved a significant milestone by installing the cobalt-60 unit Eldorado A, making it the first in the entire Southeast Asia region to do so. This was a groundbreaking step in radiation therapy, setting a precedent for cancer treatment in the region.

The same year witnessed another pioneering achievement with the establishment of the first nuclear medicine department in oncology, enhancing the institute's diagnostic and therapeutic capabilities. The 1960s marked the inception of the country's first pediatric oncology institute at the Adyar Cancer Institute, addressing the critical need for specialized care for young cancer patients. Under her visionary leadership, 1971 saw the creation of the first medical oncology unit and blood component unit, further expanding the institute's comprehensive care offerings.

Continuing its trajectory of innovation, in 1976, the institute installed India's first linear accelerator (LINAC), a major advancement in delivering precise radiation therapy. Her relentless commitment to education and training led to the establishment of the nation's first three-year postdoctorate programs in medical oncology and surgical oncology in 1984, setting new standards for oncology education and expertise.

The turn of the millennium saw yet another pioneering achievement with the establishment of India's first hereditary cancer clinic in the year 2000, providing critical insights and care for those at genetic risk of cancer. Each of these milestones not only reflects the institute's commitment to advancing cancer care but also underscores her indomitable spirit and relentless pursuit of excellence, which continues to inspire generations of oncologists and healthcare professionals.

Leadership role in oncology and public health

Her leadership in oncology and public health was marked by a remarkable range of influential roles and enduring contributions. Serving as the director of the Cancer Institute for over two decades, she played a pivotal role in shaping the institute's vision and mission, driving it toward excellence in cancer care and research.

Her expertise was sought after on both national and international platforms. She served on the World Health Organization's Advisory Committee on Cancer until March 2005, where she contributed to global cancer control strategies [6]. Her involvement extended to the Tamil Nadu State Planning Commission for Health, where she played a key role in shaping health policies and was the convener of the State Advisory Board on Cancer, ensuring that cancer care remained a priority in public health planning.

She was also the chairman of the INDO-US Collaborative Group on Lymphoid Neoplasias (Indian Chapter), where she facilitated important research collaborations between India and the United States. Her commitment to advancing medical research was further reflected in her membership in various Indian Council of Medical Research (ICMR) committees, including the ICMR Task Force on Registries, which played a crucial role in establishing cancer registries across India. Additionally, she was a member of the Syndicate of Anna University, contributing to the academic discourse in health sciences.

Her leadership extended to professional organizations, where she served as the president of the Indian Society of Oncology from 1988 to 1990 and the president of the Asian and Pacific Federation of Organizations for Cancer Control from 1997 to 1999. She was also an expert in the group addressing public concerns related to the Kudankulam nuclear power project, where her expertise helped dispel fears and provided reassurance on public health issues.

Through these diverse roles, she not only influenced the course of cancer treatment and research in India but also contributed significantly to global health initiatives, leaving an indelible mark on the field of oncology and public health.

Healing with heart: Advocacy for patient-oriented care

Advocating for compassionate care was central to her philosophy as a medical professional. She firmly believed that a physician's duties extended far beyond administering treatment, and she championed the idea that empathy and understanding were as vital as clinical expertise. Her approach to healthcare insisted on the importance of holistic, patient-oriented care, integrating the emotional and psychological well-being of patients with their physical treatment [7]. She developed and implemented protocols that reflected this ethos, ensuring that the patient's overall experience was always important in their journey through cancer care.

Her unwavering commitment to patient welfare was evident in many instances, but one particular example stands out. During a time when cancer drugs were not manufactured in India, patients had to rely on expensive imports from the United States. Due to a 100% customs tax, these essential medications were priced out of reach for many Indian cancer patients, compounding their financial burdens during an already difficult time. Despite facing bureaucratic hurdles, she tirelessly advocated for the removal of this tax, knowing about the fact that it imposed immense injustice on those in need of lifesaving treatment.

Her persistent efforts eventually led her to appeal directly to the then Finance Minister of India, Mr. Yashwantrao Chavan. She eloquently explained the financial hardships and the inequity faced by Indian cancer patients due to the prohibitive cost of these drugs. Moved by her dedication and the compelling case she presented, Mr. Chavan was persuaded to take action. In a significant victory for cancer patients across the country, he decided to abolish the customs tax on cancer drugs, making them more affordable and accessible. This act not only alleviated a significant financial burden but also underscored her unwavering commitment to advocating for her patients' rights and well-being.

Her legacy in advocating compassionate care remains a testament to her belief that healthcare is as much about humanity as it is about medicine.

Transforming rural healthcare: A visionary approach to cancer screening

Her groundbreaking contribution to the development of India's first major cancer survey and early detection program in rural areas has been widely revered. Understanding the critical need for early diagnosis in improving cancer outcomes, she spearheaded initiatives that brought lifesaving cancer screening to the most underserved populations in the country.

One of her most impactful efforts was the launch of a comprehensive program aimed at the early detection of cancer, particularly targeting cervical cancer, which remains one of the leading causes of cancer-related deaths among females in India. Identifying the barriers to healthcare access in rural areas, she implemented a program that trained hundreds of village nurses to screen females for cervical cancer [8]. This initiative not only empowered local healthcare workers with the skills and knowledge needed to conduct vital screenings but also brought critical healthcare services directly to the doorstep of rural communities.

Her work in this area was transformative, as it addressed the challenges of healthcare delivery in remote and resource-limited settings. By equipping village nurses with the tools and training necessary for effective cancer screening, she significantly increased the reach and impact of early detection efforts, leading to earlier diagnosis and better treatment outcomes for countless females.

This program also served as a model for other public health initiatives, demonstrating the effectiveness of community-based interventions in combating cancer in rural areas. Her efforts laid the foundation for future cancer control programs in India and underscored the importance of accessible, community-oriented healthcare services in reducing cancer mortality rates across the country.

Setting benchmarks: The evolution of India's premier cancer registry

Data-driven healthcare was a cornerstone of her approach to advancing cancer treatment and research. After having known the crucial role of accurate data in understanding and combating cancer, she successfully persuaded the Indian Council of Medical Research (ICMR) to establish demographic registries across India. This initiative led to the creation of three ICMR-sponsored cancer registries in Bombay, Bangalore, and Madras in 1982 [9]. These registries, by 1984, had collected critical data that would later form the backbone for the formulation of the National Cancer Control Project in 1986, a major milestone in India's fight against cancer.

One of the most distinguished features of the Cancer Institute is its cancer registry, which includes both a hospital-based and a demographic registry. These registries are not only considered the best in India but also are regarded as benchmarks for cancer data collection and management. The meticulous documentation, systematic storage, and efficient retrieval of case sheets are unparalleled in the country, ensuring that patient information is both comprehensive and accessible. Remarkably, the institute boasts a follow-up rate exceeding 90%, a record in the Indian healthcare context, reflecting the institute's commitment to long-term patient care and data accuracy.

Her foresight in establishing these registries has significantly advanced the understanding of cancer patterns, patient outcomes, and survival rates in India. The institution she led became a place of pilgrimage for those interested in cancer data management, showcasing the magnitude and effectiveness of the registry system she helped create.

In addition to its registry, the institute also houses a preventive oncology division, which plays a vital role in raising cancer awareness, providing public education, and promoting early detection strategies. Her leadership extended beyond clinical care to encompass large-scale public health initiatives, such as the Tamil Nadu Cancer Registry Project. This massive population-based surveillance study covers the entire state of Tamil Nadu, home to 80 million people, making it the largest cancer registry in the world by population coverage. Under her mentorship, this project has become a model for cancer surveillance, offering invaluable insights into cancer epidemiology in India [10].

Academician

Dr. Shanta cherished her role as a teacher. Following a visit to the Mayo Clinic in the United States during the 1970s, she methodically set up distinct medical and pediatric oncology departments at the center. Recognizing the poor prognosis of patients with acute lymphoblastic leukemia, she and Dr. Magrath established the "MCP 841 protocol," which was used by other institutions worldwide [11]. Dr. Krishnamurthi played a key role in the establishment of other specialties, as well as a dedicated surgical oncology unit. Her efforts led to the establishment of a new era in Indian medical oncology (Doctorate of Medicine {DM}) and surgical oncology (Master of Chirurgae {MCh}) education.

Having published more than 95 articles in both domestic and foreign publications, written numerous chapters for cancer books, given numerous esteemed speeches, and taken part in numerous national and international conferences, she made an effort to change how the general public saw cancer, focusing in particular on the widespread dejection and anxiety that go along with it. She was so critical of the perception of cancer among society; she used to say that the C word in cancer also symbolizes cure, care, and compassion [12]. She was particularly critical of the metaphorical application of the term "cancer" to circumstances that are dangerous, unmanageable, or dismal [13].

Awards and recognition

**Figure 1:**
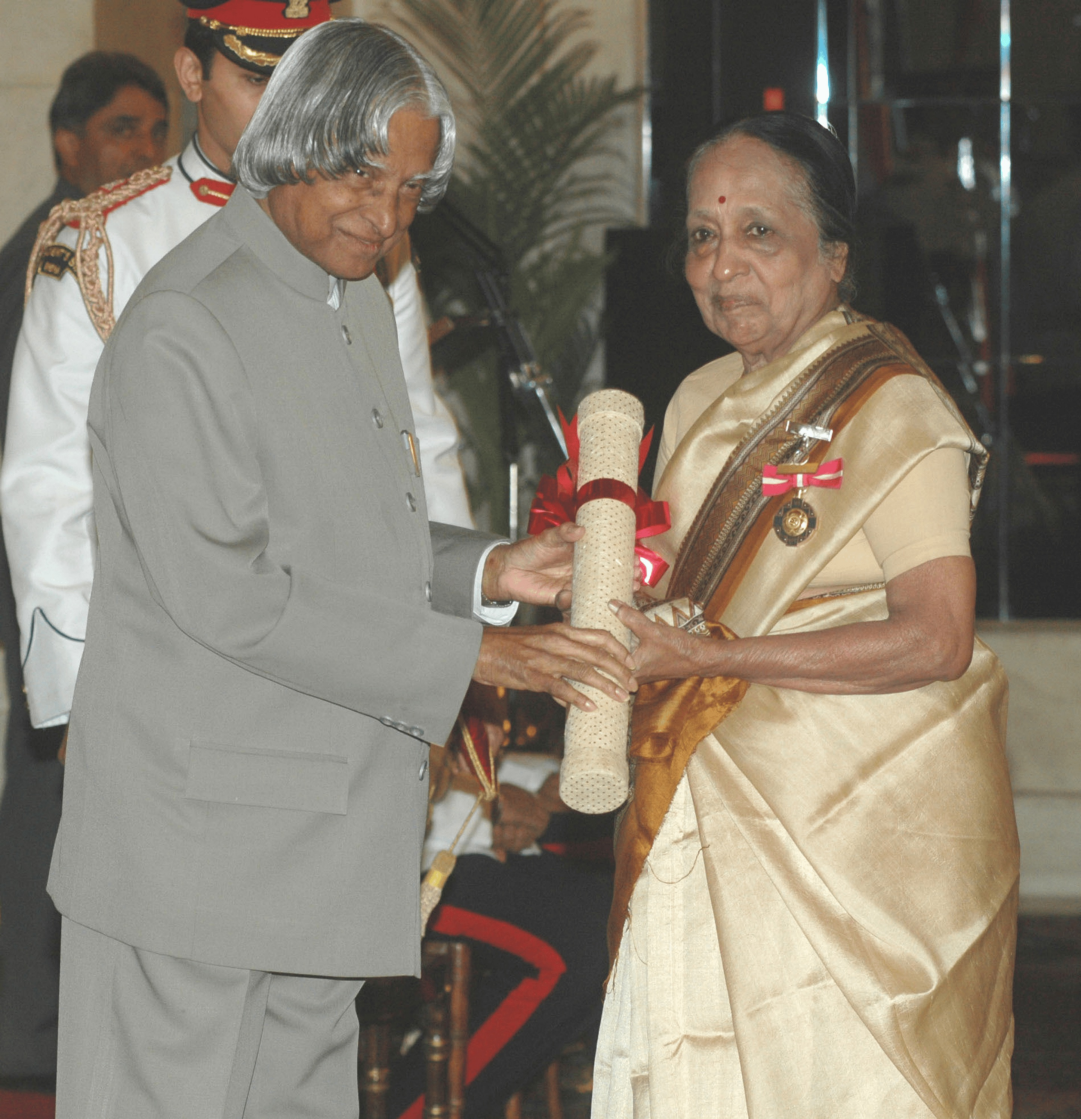
Dr. Shanta bestowed with the Padma Bhushan in 2006

**Figure 2:**
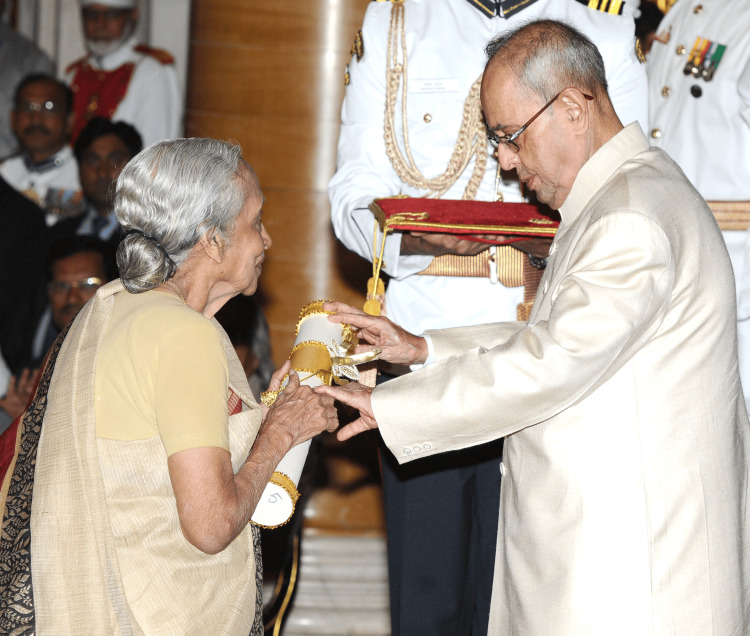
Dr. Shanta bestowed with the Padma Vibhushan in 2016

Awarded the Padma Shri Award in 1986, the International Association of Cancer Registries Award for work on developing registries in India in 1997, and the Nazli Gad-el-Mawla Award for cancer control in a resource-poor country in Brussels in 2002, Dr. V. Shanta is an elected fellow of the National Academy of Medical Sciences. She was bestowed with India's highest civilian honors, the Padma Bhushan in 2006 (Figure 1) and the Padma Vibhushan in 2016 (Figure 2) [14], after winning the Ramon Magsaysay Award in 2005, Asia's top prize, which she dedicated to her institute [12,15].

Message to the younger generation

Her vision for the younger generation was as follows: "The younger generation must ensure that the wonderful technologic advances they have are to be used not because they are available, but because they add value and are cost-effective. They must participate in areas where there is social injustice in healthcare; express their opinion without fear, where corruption exists and where change is needed. Take important decisions for your patients as you would take for your dear ones" [10].

## Conclusions

Dr. V. Shanta's life and career of nearly 65 years were marked by an unwavering commitment to advancing cancer care in India. Her legacy is a multifaceted masterpiece. As a clinician, she was a beacon of hope, offering solace and expertise to countless patients. As an administrator, she built an institution that stands as a global benchmark for cancer care. As a researcher, she pushed the boundaries of knowledge, laying the groundwork for future breakthroughs. As a social reformer, she challenged societal norms, ensuring that cancer care was accessible to the most marginalized. For the medical community, Dr. Shanta is an enduring inspiration. Her life story is a clarion call to prioritize patient-centric care, to innovate relentlessly, and to never underestimate the power of human compassion. As we stand on the shoulders of this giant, we are challenged to carry forward her legacy by pushing the frontiers of cancer research, expanding access to care, and fostering a culture of hope and healing. Her life and work have enriched our nation and inspired the world. For in the legacy of Dr. V. Shanta, we find not just a blueprint for cancer care but also a roadmap for a healthier, more compassionate society.

"In the end, it's not the years in your life that count. It's the life in your years." - Abraham Lincoln
